# Non-invasive measurement using cardiovascular magnetic resonance of changes in pulmonary artery stiffness with exercise

**DOI:** 10.1186/s12968-015-0213-2

**Published:** 2015-12-13

**Authors:** Omid Forouzan, Jared Warczytowa, Oliver Wieben, Christopher J. François, Naomi C. Chesler

**Affiliations:** Department of Biomedical Engineering, University of Wisconsin-Madison, Engineering Centers Building, 1550 Engineering Drive, Madison, WI 53706 USA; Department of Medical Physics, Wisconsin Institutes for Medical Research, 1111 Highland Avenue, Madison, WI 53705-2275 USA; Department of Radiology, University of Wisconsin, School of Medicine and Public Health, E3/366 Clinical Science Center, 600 Highland Avenue, Madison, WI 53792-3252 USA

**Keywords:** Cardiovascular magnetic resonance, Exercise stress, Arterial stiffness, Pulmonary artery distensibility, Pulse wave velocity, Pulmonary hypertension

## Abstract

**Background:**

Exercise stress tests are commonly used in clinical settings to monitor the functional state of the heart and vasculature. Large artery stiffness is one measure of arterial function that can be quantified noninvasively during exercise stress. Changes in proximal pulmonary artery stiffness are especially relevant to the progression of pulmonary hypertension (PH), since pulmonary artery (PA) stiffness is the best current predictor of mortality from right ventricular failure.

**Methods:**

Cardiovascular magnetic resonance (CMR) was used to investigate the effect of exercise stress on PA pulse wave velocity (PWV) and relative area change (RAC), which are both non-invasive measures of PA stiffness, in healthy subjects. All 21 subjects (average age 26 ± 4 years; 13 female and 8 male) used a custom-made MR-compatible stepping device to exercise (two stages of mild-to-moderate exercise of 3–4 min duration each) in a supine position within the confines of the scanner. To measure the cross-sectional area and blood flow velocity in the main PA (MPA), two-dimensional phase-contrast (2D-PC) CMR images were acquired. To measure the reproducibility of metrics, CMR images were analyzed by two independent observers. Inter-observer agreements were calculated using the intraclass correlation and Bland-Altman analysis.

**Results:**

From rest to the highest level of exercise, cardiac output increased from 5.9 ± 1.4 L/min to 8.2 ± 1.9 L/min (*p* < 0.05), MPA PWV increased from 1.6 ± 0.5 m/s to 3.6 ± 1.4 m/s (*p* < 0.05), and MPA RAC decreased from 0.34 ± 0.11 to 0.24 ± 0.1 (*p* < 0.05). While PWV also increased from the first to second exercise stage (from 2.7 ± 1.0 m/s to 3.6 ± 1.4 m/s, *p* < 0.05), there was no significant change in RAC between the two exercise stages. We found good inter-observer agreement for quantification of MPA flow, RAC and PWV.

**Conclusion:**

These results demonstrate that metrics of MPA stiffness increase in response to acute moderate exercise in healthy subjects and that CMR exercise stress offers great potential in clinical practice to noninvasively assess vascular function.

## Background

Exercise stress tests, in which a subject’s heart rate and cardiac output (CO) are increased by physical exercise, are often conducted clinically to monitor the functional state of the heart and vasculature. When non-invasive imaging is performed in conjunction with exercise stress, echocardiography and single photon emission computed tomography (SPECT) are most often used to assess parameters such as ventricular function and exercise-induced hemodynamic changes. However, echocardiography and SPECT are suboptimal for imaging the pulmonary circulation. Echocardiography of the pulmonary circulation is challenging due to the difficulties in obtaining an acceptable acoustic window, aligning the ultrasound beam with the flow direction [1] and short penetration depth [2]. In contrast, cardiovascular magnetic resonance (CMR) is capable of obtaining anatomical data at arbitrary imaging planes and measuring flow in any direction at the expense of longer scan times. CMR stress tests are typically conducted with pharmacological stress due to limited space for physical activity inside the scanner bore and sensitivity to motion artifacts. However, the stress condition induced by physical exercise better represents cardiovascular stresses experienced in daily life.

Devices that allow exercise stress tests to be performed within conventional MRI systems [3] and open MRI systems [4] have recently been developed. Using these systems to quantify the effect of acute exercise on hemodynamics, and the functional response of the pulmonary circulation to exercise stress, may offer new, clinically useful insight into pulmonary hypertension (PH) progression [5, 6]. For example, decreased proximal pulmonary artery (PA) elasticity has considerable pathophysiological implications in PH. Elasticity is a measure of an artery’s ability to expand and recoil with each contraction of the heart; large, proximal artery elasticity buffers the hemodynamic impact of the pulsatile flow and ensures constant flow to distal tissues. By elevating right ventricular (RV) afterload, PA stiffness can suppress RV contractile performance and ultimately decouple the RV from its vascular load [7, 8]. Also, by increasing downstream blood flow pulsatility, decreased PA elasticity (or increased PA stiffness) can enhance pulmonary arteriolar inflammation [9]. Observations of increased PA stiffness in the early stages of PH suggest its potential role in the development and progression of the disease [8].

A non-invasive metric of main PA (MPA) area strain over the cardiac cycle, the relative area change (RAC), has been shown to correlate with MPA stiffness and predict mortality in patients with PH [10–12]. Pulse wave velocity (PWV), which is a measure of wave-propagation velocity in the major arteries, is another non-invasive correlate of PA stiffness [13–15]. Higher PWV in the systemic circulation is considered as a marker of increased cardiovascular risk [16]. Both MPA RAC and MPA PWV can be measured non-invasively with CMR [12, 15].

In this study, we investigated the effect of exercise stress on non-invasive, CMR metrics of MPA stiffness. Based on evidence that PA stiffness can increase acutely as a consequence of an acute elevation in pulmonary artery pressure [17, 18], our hypothesis was that acute exercise will decrease RAC and increase PWV in young healthy subjects.

## Methods

### Subjects and exercise protocol

**Fig. 1:**
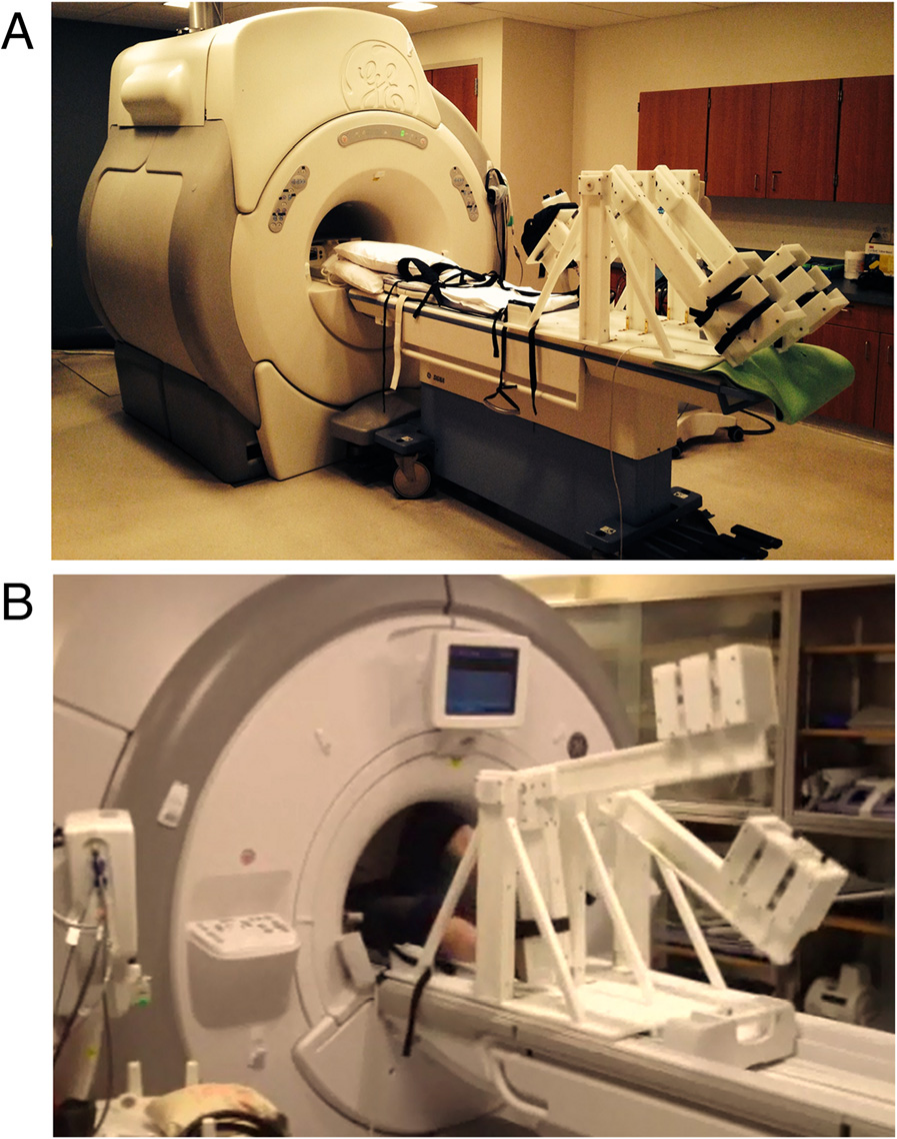
CMR-compatible stepper exercise device shown in use in a CMR scanner. **a** Photograph of the CMR-compatible stepper exercise device on the bed of the GE 1.5 T CMR scanner. **b** Photograph of the CMR-compatible stepper exercise device while in-use. The subject is exercising in the supine position while his/her torso, which is being imaged, is inside the MR bore. As the subject extends and then flexes alternating knees, the L-shaped lever arms are raised and then lowered in a dynamic stepping motion. The stepping cadence is measured by the built-in motion sensor which is connected to a computer in the monitoring room

This study was approved by the Institutional Review Board and was compliant with the Health Insurance Portability and Accountability Act. A total of 21 volunteers aged 26 ± 4 year old (13 females and 8 males) were recruited, following written informed consent and undergoing screening with CMR safety questionnaires. All subjects were free of overt cardiovascular, pulmonary and renal disease. Exercise stress was conducted using a custom-made MR-compatible stepping device that allows subjects to exercise with a leg-stepping motion while their torso is confined within an MR bore and MR imaging can start immediately after the completion of exercise (Fig. 1) [3]. The device is equipped with electronics that measure stepping cadence real-time; a metronome assists subjects in maintaining a set cadence. The exercise workload was adjusted by controlling the cadence and increasing workload per step. After acquiring images at rest, subjects performed 2 stages of mild-to-moderate exercise (target workloads of ~35 and 45 W) of 3–4 min duration each. To capture consistent images and minimize the motion artifact, images were acquired during a brief cessation from exercise.

### CMR

**Fig. 2:**
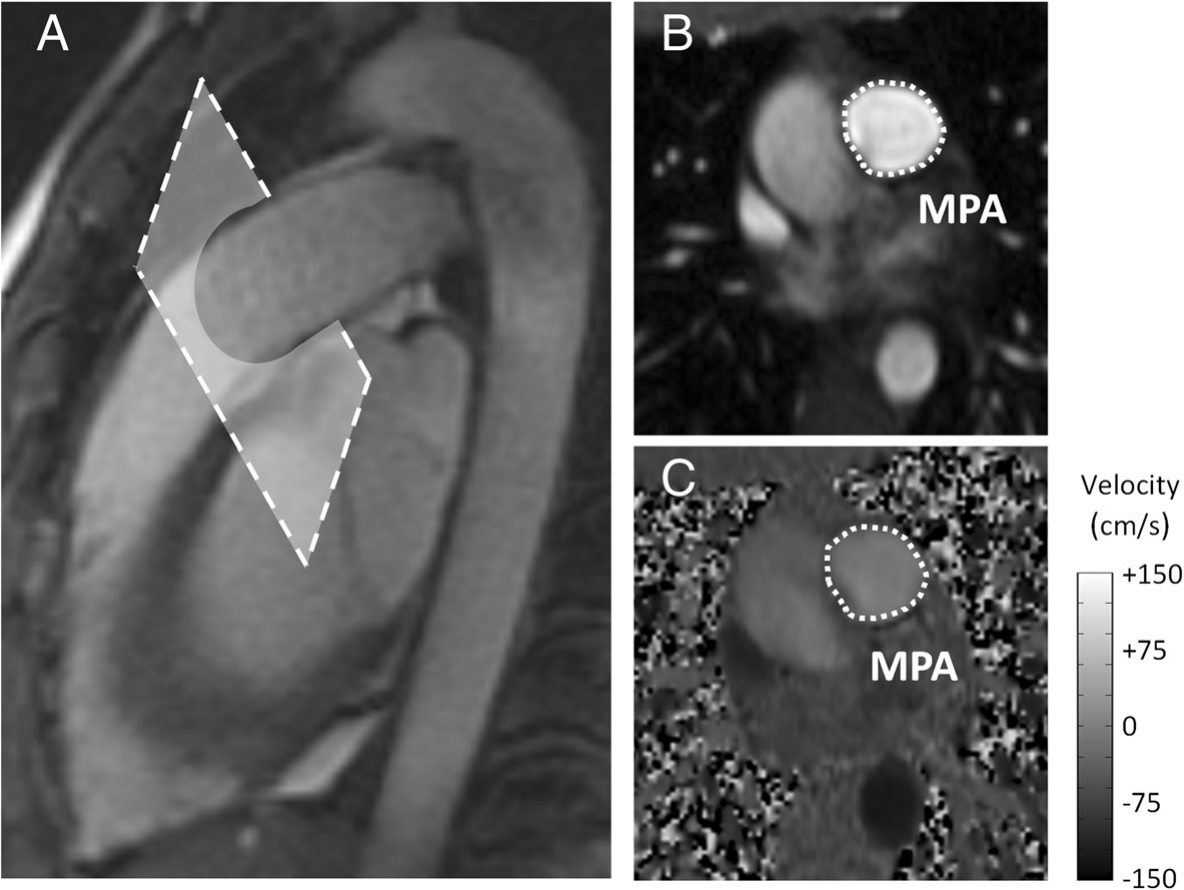
MR images of main PA for a representative subject. **a** Sagittal image of the MPA displaying the location of 2D-PC acquisition slice. The imaging plane is prescribed in a double-oblique plane perpendicular to the flow direction. **b** 2D-PC magnitude image of the main PA, used to define and measure the cross-sectional area. **c** 2D-PC phase image, used to calculate the blood flow velocity, of the MPA. Grayscale-bar represents flow velocity in the phase image (ranges from -150 cm/s to 150 cm/s)

CMR was performed on clinical 1.5 T scanners (HDxt, GE Healthcare, Waukesha, WI and GE Healthcare Optima MR450W, Waukesha, WI) using an 8-channel cardiac coil and vector electrocardiographic (ECG) gating. Two-dimensional, velocity encoded phase contrast (2D-PC) images were acquired in double oblique planes through the MPA distal to the pulmonic valve with the imaging plane orthogonal to the blood flow direction (Fig. 2). Image parameters for 2D-PC were: 35 × 26 cm field of view, supine position - head first, 256 × 160 acquisition matrix (reconstructed to 256 × 256), 7 mm slice thickness, ±62.5 kHz bandwidth, 150 cm/s velocity encode (“venc”), TR/TE = 5.5/2.6 milliseconds (full echo), prospective electrocardiographic gating with an acquisition window of 10 ms, k-space segmentation factor of 8, and parallel imaging (ASSET) with an acceleration factor of 2. Data were reconstructed into 40 time frames throughout the cardiac cycle. Imaging was acquired while suspending ventilation for approximately 15–17 s depending on the actual heart rate.

### CMR data analysis

**Fig. 3:**
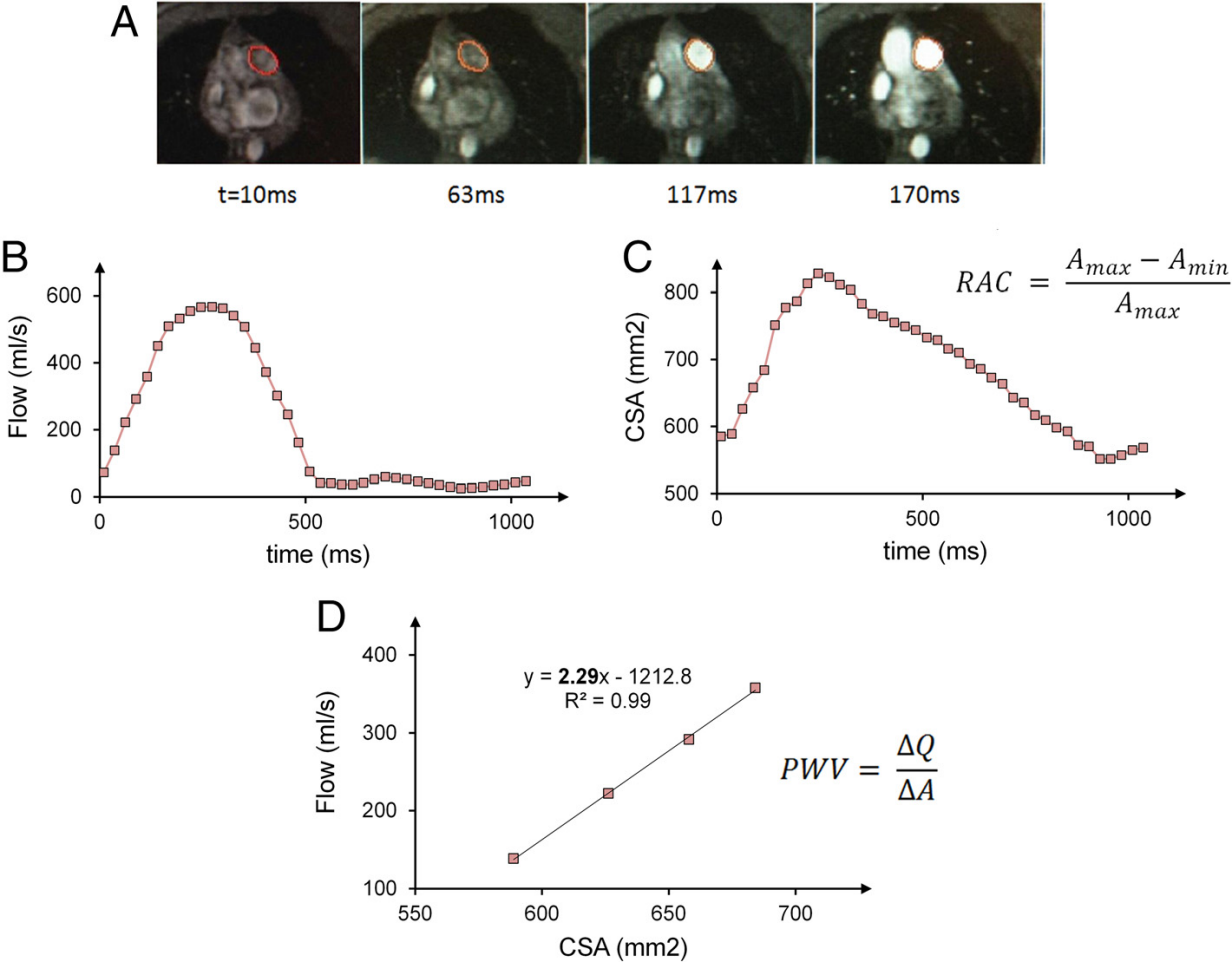
RAC measurement and PWV measurement with flow-area method for a representative subject. **a** Time-lapse of magnitude and phase images of main PA cross-section during early systole. **b** and **c** Flow rates and cross-sectional area (CSA) of the main PA were assessed based on the contours shown in the images. RAC is measured using maximum and minimum CSA throughout the cardiac cycle **d** Flow and CSA are measured from the phase contrast-derived velocity maps integrated over the magnitude-derived area. PWV velocity is calculated as the slope of QA plot at early systole

2D-PC images were analyzed using the GE flow quantification software “CV Flow version 3.3” (GE Healthcare, Milwaukee, WI). Two independent observers (observer 1 and observer 2) performed data analysis using the same software. To obtain blood flow velocity and CSA, the contours of the MPA were outlined manually (Fig. 3a). Flow rate was calculated as the product of the CSA and the mean velocity (Fig. 3b). Stroke volume was calculated as the average flow rate over the cardiac cycle (cardiac output; CO) divided by the heart rate. Relative area change (RAC) was calculated as (max cross-sectional area (CSA) – min CSA)/max CSA) (Fig. 3c) [12].

PWV is typically obtained by measuring the travel-time of the flow waves between two specific locations separated by a known distance (transit-time method) [19]. However, using this method to measure PWV in the MPA is challenging due to the short vessel length and limited temporal resolution. Therefore an alternative method based on measuring flow and area (QA method) at one location has been introduced [13]. Specifically, PWV was calculated from the slope of the line fitted to the flow-area data, which represents the ratio of flow to area changes (∆Q/∆A), during early systole (Fig. 3d) [13].

### Statistical analysis

All data are reported as mean value ± standard deviation. Statistical analysis was performed using R software (Foundation for statistical computing, version 3.0.1). The association between hemodynamic parameters and exercise condition was analyzed using a linear mixed-effect model with repeated measures (generalized least squares). Tukey’s honestly significant difference test was used as a post-hoc test of significance. Inter-observer agreement for each of the metrics was evaluated by calculating the intraclass correlation coefficient and respective confidence interval at 95 %. A *p*-value < 0.05 was considered evidence of statistical significance.

## Results

Twenty-one subjects performed supine exercise and were successfully imaged with CMR. All study participants completed CMR protocols at rest and for two exercise stages, with no premature termination of tests. After analyzing all CMR data, 6 subjects were excluded from the final analysis because their images were not readable mainly due to shifted acquisition planes and motion artifacts. As a result, the final analyzed study group consisted of 15 subjects (26.5 ± 3.9 years old, 9 females and 6 males). The workloads achieved in the two stages of exercise were 35.1 ± 8.2 W and 45.7 ± 10.8 W.

**Fig. 4:**
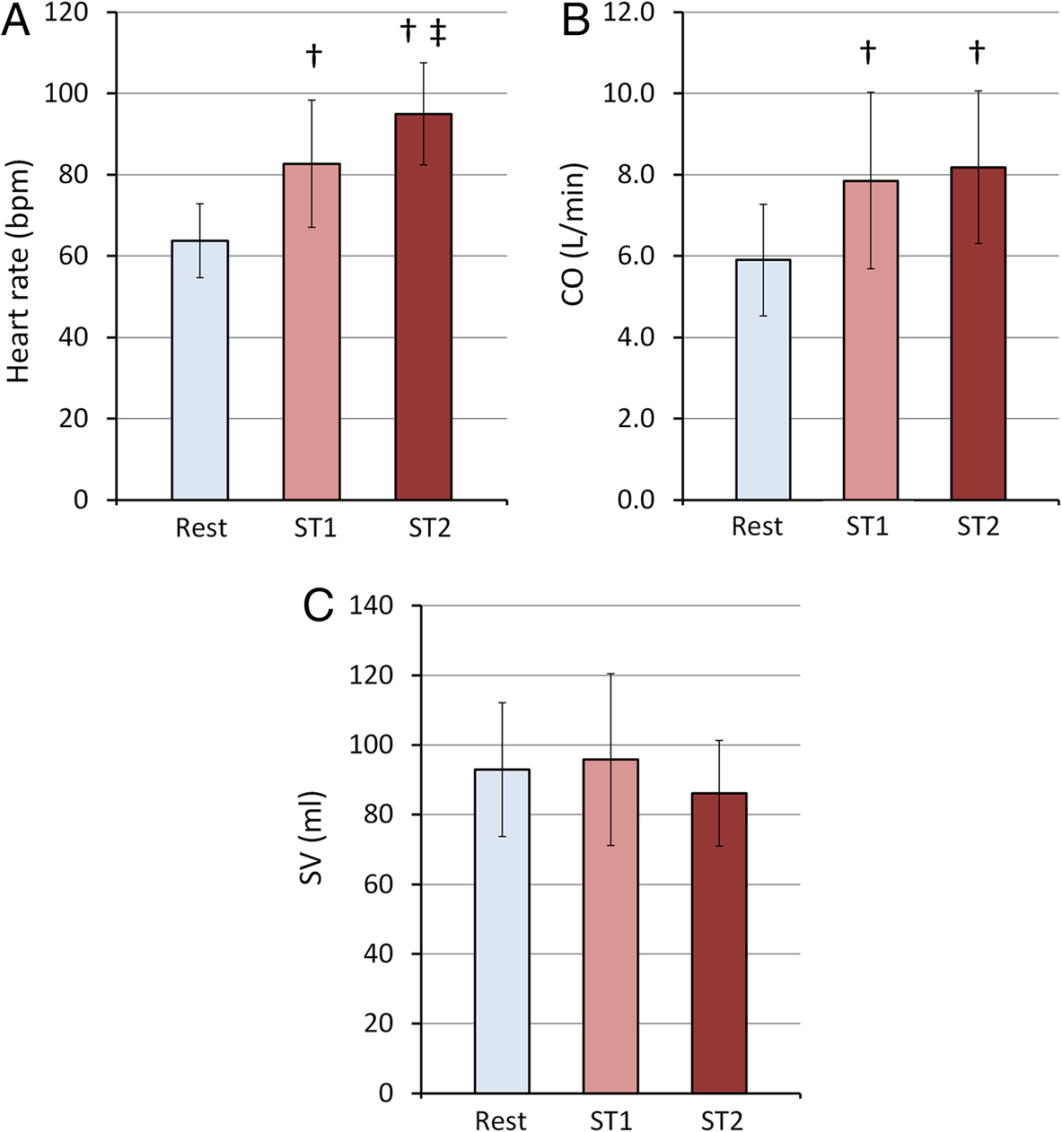
Summary of hemodynamics data. Averaged values of Heart rate (**a**), Cardiac output (**b**) and Stroke volume (**c**) at rest and with each stage of physical exercise averaged for 15 subjects. Rest, ST1 and ST2 represent data acquired at rest, with 35 W and 45 W exercise respectively. Statistical analysis was performed using a one-way ANOVA with repeated measures and Tukey’s test for multiple comparisons. †*P* < 0.05, vs. Rest; ‡*P* < 0.05, vs. ST1. The data in the bar graph represent the mean ± SD

Heart rate and CO significantly increased following the first stage of exercise (ST1, 35 W) by 30 % and 33 % (Fig. 4a & b). At the second stage of exercise (ST2, 45 W) heart rate further increased significantly by 15 % leading to a total increase of 50 % compared with rest. There was no significant difference between the measured CO at ST2 vs. ST1. SV did not change with either exercise stage (Fig. 4c).

**Fig. 5:**
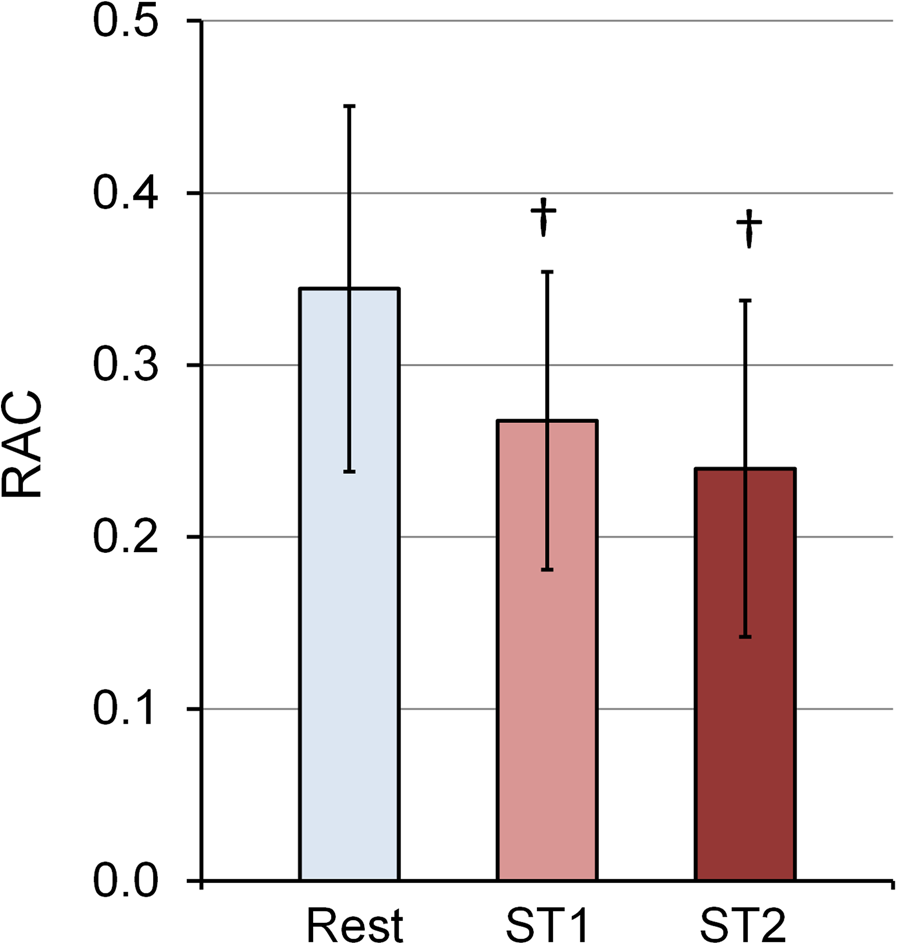
Effect of different exercise-stress stages on MPA-RAC. Average RAC data from 15 subjects at different stress conditions indicates lower RAC is associated with higher exercise workload (ST1 = 35 W, ST2 = 45 W). †*P* < 0.05, vs. Rest. The data in the bar graph represent the mean ± SD

MPA RAC decreased from 0.34 ± 0.11 at rest to 0.27 ± 0.09 at ST1 and to 0.24 ± 0.10 at ST2; there was no significant difference between RAC for ST1 vs. ST2 (Fig. 5).

**Fig. 6:**
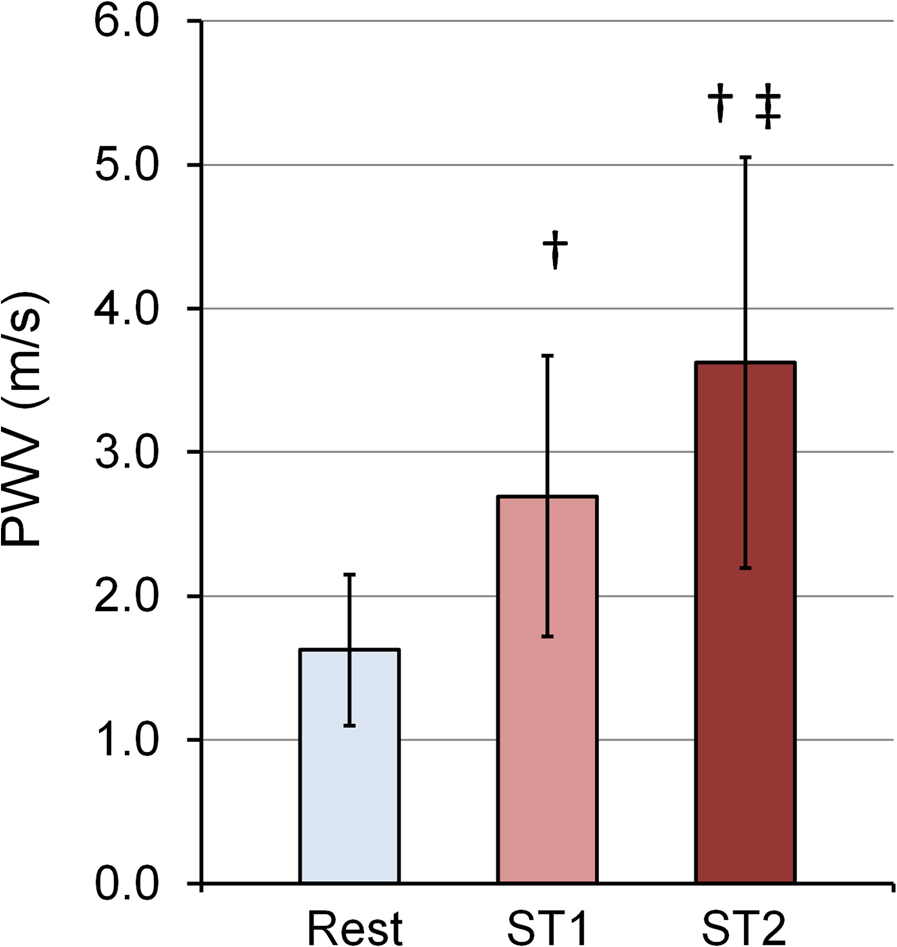
Effect of different exercise-stress stages on MPA-PWV. Averaged PWV data for different stress conditions indicating higher PWV associated with higher exercise workload (ST1 = 35 W, ST2 = 45 W) (*n* = 15). †*P* < 0.05, vs. Rest; ‡*P* < 0.05, vs. ST1. The data in the bar graph represent the mean ± SD

MPA PWV increased with exercise (Fig. 6). There was a significant increase in PWV with exercise from 1.6 ± 0.5 m/s at rest to 2.7 ± 1.0 m/s at ST1 and 3.6 ± 1.4 m/s at ST2 (average *R*^2^ = 0.94, *p* < 0.05).

**Fig. 7:**
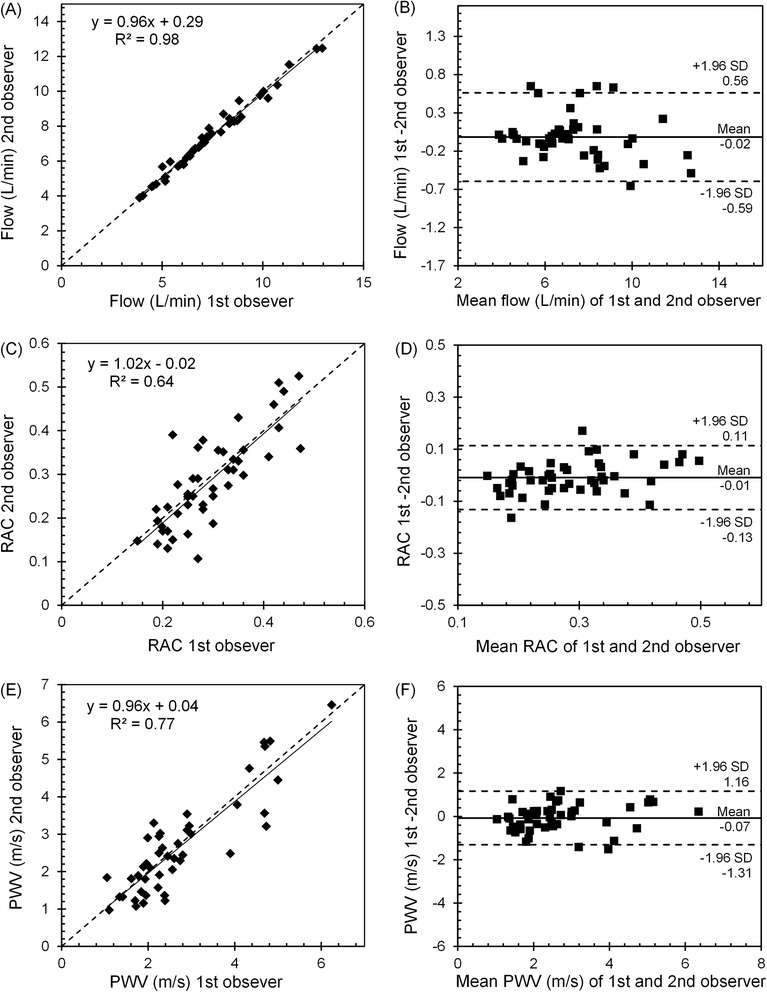
Intraclass correlation plot and Bland-Altman plot for inter-observer agreement. Intraclass correlation plot and Bland-Altman plot for MPA flow (**a** & **b**), RAC (**c** & **d**), PWV (**e** & **f**). Solid lines on the correlation plots represent fitted linear regression and the dashed line represents the identity line. On the Bland-Altman plots, solid and dashed lines show the mean difference and the 95 % limits of agreement (±1.96SD), respectively

The reproducibility in the evaluation of the MPA flow showed a good inter-observer agreement (intraclass correlation coefficient: 0.99 (95 % confidence interval [0.98, 0.99]). Figure 7a presents a dispersion correlation plot with values for MPA flow obtained by the two observers, where data seem to approximate a straight line, i.e., data regarding the MPA flow obtained by the two observers are positively correlated (*p* < 0.001). Also, the Bland-Altman analysis for MPA flow showed negligible differences between the two observers with the mean difference (bias) of −0.016 (95 % confidence interval [−0.59,+0.56]) (Fig. 7b). Similarly, the inter-observer variability analysis for RAC and PWV showed good agreement between the two observers. For RAC, intraclass correlation coefficient was 0.78 (95 % confidence interval [0.64, 0.87]) and Bland-Altman mean difference was −0.0087 (95 % confidence interval [−0.13,+0.11]) (Fig. 7c & d) and for PWV, intraclass correlation coefficient of 0.99 (95 % confidence interval [0.79, 0.93]) and mean difference of −0.07 (95 % confidence interval [−1.31,+1.16]) was observed (Fig. 7d & e).

## Discussion

The present study evaluated the effect of acute supine exercise on MPA stiffness of healthy humans using an CMR-compatible exercise device. Cardiac output and heart rate increased significantly at each stage of exercise, which indicates the effectiveness of our CMR-compatible exercise device in inducing a stress condition in young healthy adults. In addition, we observed a significant decrease in RAC and significant increase in PWV with acute exercise, both of which indicate an increase in MPA stiffness.

Stiffness of the large, conduit pulmonary arteries such as the MPA determines pulse pressure, which directly influences myocardial workload and ventricular function [7, 8] as well as downstream endothelial function [9]. MPA stiffness is also a strong predictor of mortality in PH [11, 12]. Direct measurement of MPA stiffness requires pressure measurements, which can currently only be achieved by invasive right heart catheterization. Using alternative non-invasive metrics of stiffness would be desirable for the clinically indicated frequent follow-ups or screening tests or the assessment of treatment responses. The RAC of the MPA is a non-invasive measure of proximal PA area-strain that is inversely correlated with the elastic modulus of proximal PA [18]. Also, decreased RAC is correlated with poor prognosis in PH patients [12]. PWV is another useful non-invasive metric of the elastic properties of the large arteries such as MPA [14, 15]. An advantage of both of these metrics is that they can be obtained using CMR alone, without invasive pressure measurements.

2D-PC CMR is widely accepted as an accurate method to non-invasively measure blood flow velocity and luminal CSA in the major arteries [20]. Here we obtained MPA PWV and RAC by measuring flow and CSA of the MPA in healthy young adults at rest and at stressed states. A popular method of estimating PWV by CMR is the transit time method, which is based on measuring the time of a pulse-wave travelling between two locations along the vessel [19]. The QA method, derived from the Bramwell–Hill equation [21], and first presented by Vulliemoz et al., is an alternative method to estimate local PWV [13]. The main advantage of the QA method over the TT method is that it is based on simultaneous measurement of flow and CSA at only one location. Therefore, it is more suitable for vessels with short segments where the temporal resolution of the CMR measurements becomes a severe limitation for the transit time method. MPA PWV has been measured using the QA method in adult patients [15] and children [14]. However, this is the first study that has investigated changes in MPA PWV in response to acute exercise.

Our results show significant increase in PWV and significant decrease in RAC with incremental exercise. The increase in PWV with exercise was significant for all the exercise stages. In contrast, RAC showed no significant change between the two exercise stages (Fig. 5). PWV reflects changes in flow as well as area whereas RAC (area strain) only reflects the changes in CSA. Additionally, using RAC as stiffness metric is based on the assumption that there is a strong linear relation between systolic and diastolic pressures and has been validated across wide range of MPA pressures, but not during exercise [12, 22]. The linear portion of the Q-A curve is generally accepted to be a reflection-free period [13, 15, 23, 24] such that the slope is a good absolute measure of PWV. Recently, Segers et al. argued that linearity does not preclude the presence of reflections but that, according to theoretical and experimental results, consistently using the linear portion provides a good relative measure of PWV [25]. Thus, the relative increase in PWV we found here with exercise using the Q-A method likely reflects increased MPA stiffness with exercise, which the RAC metric was not sensitive enough to capture.

**Fig. 8:**
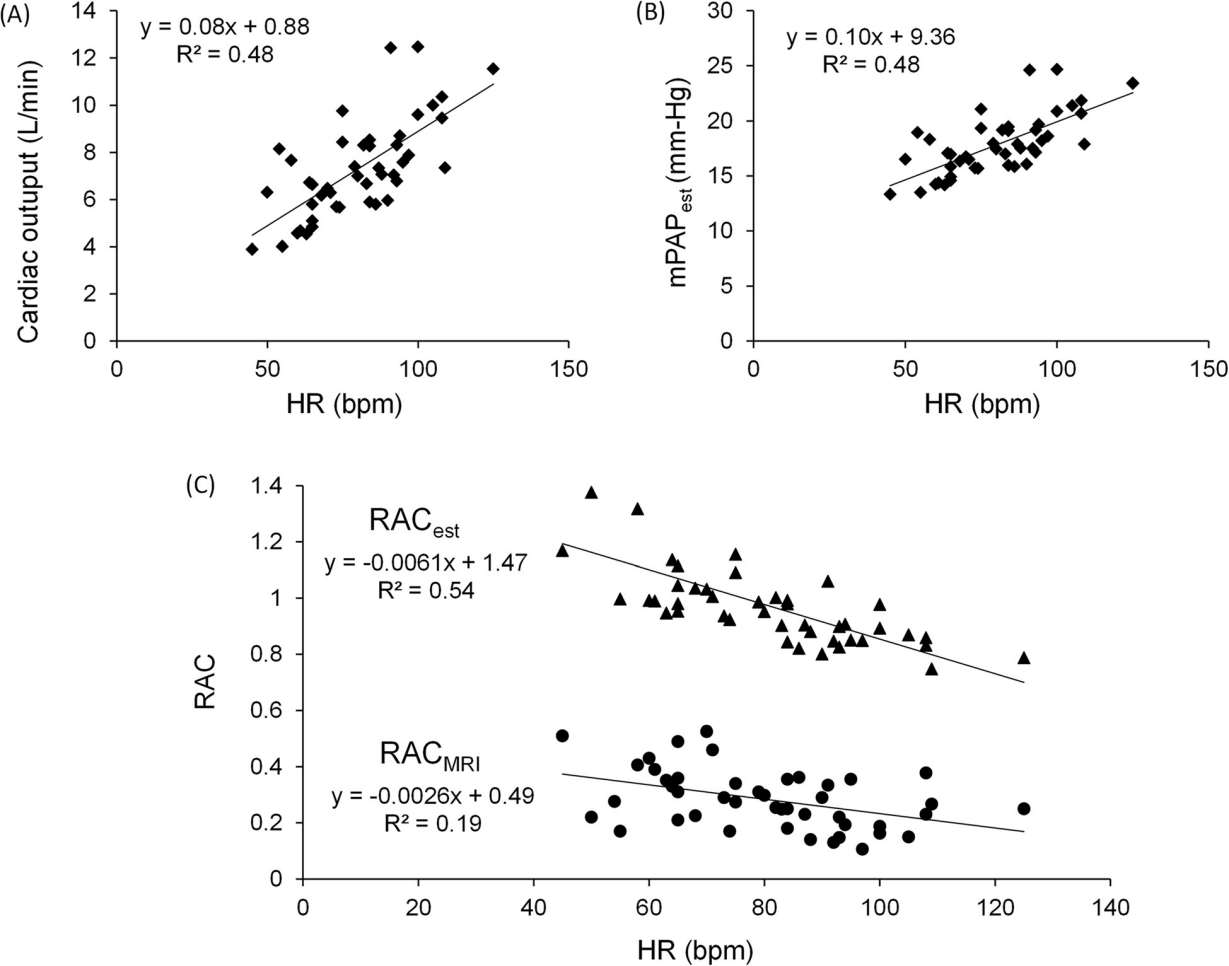
Estimating RAC based on existing empirical models. Correlation between CO and HR (as a measure of stress-state) shows increased CO with increase in HR (**a**). Estimated values of mPAP for 15 subjects as a function of HR shows increase in mPAP with increased HR (**b**). Estimated RAC_est_ for different heart rates based on the empirical model and RAC_CMR_ for different heart rates measured with exercise-CMR stress test (**c**)

It has been shown that in the absence of remodeling (i.e., wall thickening and irreversible conformational changes), acute increases in MPA stiffness can be caused by increased arterial pressure [17]. Bellofiore et al. investigated the correlation between RAC and PA pulse pressure (PAPP) in a canine model of acute embolization and proposed a linear equation; RAC = 0.1403 × (SV/PAPP) + 0.1279, where SV is stroke volume in ml and PAPP is in mm-hg [17]. To better understand the relationship between MPA stiffness and MPA pressure, we used this correlation to estimate RAC (RAC_est_) with MPA pulse pressure. However, since measuring the pressure in the PAs requires invasive methods and cardiac catheterization of healthy subjects has ethical and practical concerns, we used the available models in the literature to estimate PA pressures of healthy individuals based on CO, which can be measured non-invasively with CMR (Fig. 4b & Fig. 8a). Argiento et al. have proposed a linear relationship between CO and mean PA pressure (mPAP) for normal healthy subjects performing exercise; mPAP = 1.37 × CO + 8.2 mm-Hg [5]. Additionally, previous studies on humans and animals have shown that systolic and diastolic PA pressures (sPAP and dPAP) are linearly related to mPAP for both healthy subjects and PH patients [26, 27]. For healthy subjects, these linear relationships are expressed as sPAP = 1.5 × mPAP + 0.46 mm-Hg and dPAP = 0.71 × mPAP - 0.66 mm-Hg [26]. Therefore PAPP, which is the difference between sPAP and dPAP, can be calculated as PAPP = 0.79 × mPAP + 1.12 mm-Hg. By combining all of the above equations, RAC can be estimated with CO (L/min) and HR (bpm) as:

$$ RA{C}_{est}=\frac{140.3\kern0.5em \times \kern0.5em CO}{HR\times \left(1.0428\kern0.5em \times \kern0.5em CO\kern0.5em +\kern0.5em 7.598\right)}+0.1279 $$

We used this empirical model to calculate RAC_est_ for all analyzed subjects (*n* = 15) in all stress conditions (Rest, ST1 and ST2). The results show that the estimated mPAP (mPAP_est_) increases and RAC_est_ decreases with increased HR (Fig. 8b & c). Also, the comparison between different exercise stages shows that RAC_est_ decreases with incremental exercise (data not shown), which is similar to what was observed for the measured RAC (RAC_CMR_) (Fig. 5). Despite the consistent over-estimation of RAC_est_, the similarity in the behavior of RAC_est_ and RAC_CMR_ in response to exercise suggests that the decreased stiffness with exercise observed in our study is due to increased MPA pressure. To confirm this interpretation, direct measurement of MPA pressure under exercise stress would be required.

We noted good repeatability in the evaluation of MPA flow, RAC and PWV shown by good inter-observer agreement and low variability in the intra-class correlation and Bland-Altman analysis. Previous studies by Ibrahim et al. [15] and Poon et al. [14] have shown good reproducibility in measuring PWV using the QA method. In this study, we present good reproducibility for the two stiffness metrics, RAC and PWV, acquired at rest and with exercise stress. The results of our analysis show that PWV has stronger reproducibility compared with RAC. This could be due to the fact that PWV is related to both flow and CSA measurements while RAC is only related to CSA measurements. Both CSA and flow are measured by manually outlining the boundaries of MPA cross-section on the images. However, in general, flow measurement is less dependent to minor variations in the outlined boundaries of MPA since the high flow region is in the middle-portion of MPA cross-section, away from the MPA boundary and mostly not affected by observer variabilities. This makes flow measurement less prone to measurement error and user variability.

There are several limitations to this study. First, the stiffness metrics used (RAC and PWV) are both based on the measurement of the CSA and therefore variations in the orientation and position of the imaging plane (potentially due to motion artifacts) could introduce error. These variations in the imaging plane were partly introduced by the involuntary motion of the upper body as a result of the physical exercise. To minimize this effect, the exercise device used in this study was equipped with a body strap and handles to stabilize the upper body motion during exercise. Another cause of motion artifact, in some cases, was the inability of the subject to hold their breath during the scan immediately after exercise. As a quality control, we discarded a total of 6 data sets where at least one scan was affected by motion artifact or out of plane MPA or MPA acquisitions with oval cross-section. Real-time 2D-PC sequences would be an alternative strategy to mitigate the motion artifacts we observed in this study.

A second limitation is that the QA method assumes unidirectional wave propagation along the vessel, which is only the case when reflective waves are not present. From a fluid mechanics point of view, wave reflections occur in elastic tubes when the pressure or flow wave meets a change of admittance [28, 29]. The branching of the MPA into right and left extralobar PAs likely causes some wave reflection that could modify the pressure and flow waves measured at the MPA. Since the MPA is quite short, the time window before the first reflected waves return to the MPA is small. Therefore, temporal resolution is a crucial factor for accurate measurement of MPA PWV using the QA method. *Peng* et al. addressed this issue by increasing the number of measured time-points during early systole using two consecutive scans at different ECG trigger delay values [2]. We did not implement this method in our MR exercise-stress study because using two consecutive scans requires a longer breath-hold, which is challenging after exercise. Overall, imaging techniques with higher temporal and spatial resolutions will be necessary to overcome these limitations and improve the accuracy of PWV measurement in MPA.

## Conclusion

In the present study, we investigated the impact of acute exercise on non-invasive metrics of PA stiffness in healthy individuals. We were able to confirm our hypothesis that acute exercise increases MPA wall stiffness. Overall, this study establishes the feasibility and reproducibility of measuring exercise-induced changes in MPA stiffness via RAC and PWV using CMR. Further studies are needed to study the mechanisms of these exercise-induced changes and potential clinical implications in patients.

## Abbreviations

PH: Pulmonary hypertension
PA: Pulmonary artery
RV: Right ventricular
CMR: Cardiovascular magnetic resonance
PWV: Pulse wave velocity
RAC: Relative area change
MPA: Main pulmonary artery
2D-PC: Two-dimensional phase-contrast
CO: Cardiac output
SPECT: Single photon emission computed tomography
ECG: Electrocardiographic
CSA: Cross-sectional area
ST1: First stage of exercise
ST2: Second stage of exercise
